# Invalids and Exertive Exercise

**Published:** 1854-01

**Authors:** 


### INVALIDS AND EXERTIVE EXERCISE.

Common consumption of the lungs destroys more people than any other half dozen diseases, while perhaps a third of all who die in civilized society, do so from ailments connected with the air passages, hence whatever tends to diffuse true knowledge on the subject must be a public good. Theories are good enough in their place, but the mass of society prefers to deal in facts, in well established whole facts; these are more tangible, and the common mind can more easily grapple with them. The diseases to which the lungs and their proper appendages are liable, are Asthma, Bronchitis, Consumption, Laryngitis or Throat ail, Croup, Pleurisy, Inflammation of the Lungs, Congestion of the Lungs, Quinsy, &c. All these diseases arise from two causes:

1. Changes of temperature.
2. The failure to keep the breathing apparatus in vigorous, full, healthful operation, by a sufficient amount daily of exertive exercise in the open air. By a wise attention to this second cause of lung diseases, the former will cease to be a cause, except in occasional cases. Not only so, threatened consumption may be effectually warded off, in the vast majority of cases, by the proper adaptation of daily out-door exertive exercise to the requirements of the system, as indicated by the condition of the pulse, the heart, the breathing organs, of which the physician ought to be the standing judge. For, when a man is an invalid, the amount of food, air and exercise requires as much of medical intelligence, experience and skill, as would the judicious exhibition of medicine.

